# Do anticodons of misacylated tRNAs preferentially mismatch codons coding for the misloaded amino acid?

**DOI:** 10.1186/1471-2199-11-41

**Published:** 2010-05-28

**Authors:** Hervé Seligmann

**Affiliations:** 1 Department of Evolution, Systematics & Ecology, The Hebrew University of Jerusalem, 91904 Jerusalem, Israel; 2 Center for Ecological & Evolutionary Synthesis, Department of Biological Sciences, University of Oslo, Blindern, N- 0316 Oslo, Norway

## Abstract

**Background:**

Accurate amino acid insertion during peptide elongation requires tRNAs loaded by cognate amino acids and that anticodons match codons. However, tRNA misloading does not necessarily cause misinsertions: misinsertion is avoided when anticodons mismatch codons coding for misloaded amino acids.

**Presentation of the hypothesis:**

Occasional compensation of misacylation by codon-anticodon mismatch necessarily occurs. Putatively, occasional error compensation may be enhanced beyond the random combination of independent errors in tRNA loading and codon-anticodon interactions: tRNA misacylation might alter potentials for codon-anticodon mismatches, perhaps specifically increasing potentials for mismatching those codons coding for the misacylated non-cognate amino acid. This hypothetical phenomenon is called 'error coordination', in distinction from 'error compensation' that assumes independence between misacylation and mismatch.

**Testing the hypothesis:**

Eventually, the hypothesis should be tested for each combination of amino acid misacylation and codon-anticodon mismatch, by comparing stabilities or frequencies of mismatched codon-anticodon duplexes formed by tRNAs loaded by their cognate amino acid with stabilities formed by that tRNA when misloaded with the amino acid coded by the mismatched codon. Competitive mismatching experiments between misloaded and correctly loaded tRNAs could also be useful, yet more sophisticated experiments.

**Implications of the hypothesis:**

Detecting error coordination implies estimating error compensation, which also promotes protein synthesis accuracy. Hence even in the absence of evidence for error coordination, experiments would yield very useful insights into misacylation and mismatch processes. In case experiments consider post-transcriptional RNA modifications (especially at wobble positions), results on codon-anticodon mismatches would enable significant improvements and sophistications of secondary structure prediction softwares. Positive results would show that protein translation enhances accuracies of products, not of single steps in the production. Ancient translational machineries putatively optimized error coordination, especially before tRNA editing by tRNA synthetases evolved: few primitive, but functionally versatile tRNA species perhaps executed low accuracy translation. Systems artificially designed/selected for low complexity and high efficiency could make use of this property for anticodons with high levels of error compensation and coordination.

## Background

The two major groups of tRNA synthetases, class I and II, seem to minimize impacts of misinserted amino acids in protein sequences by tRNAs that were misloaded by these tRNA synthetases [1,2]. Accurate loading of tRNA acceptor stems with cognate amino acids by tRNA synthetases is a crucial step in protein synthesis, and indeed misacylated (misloaded) tRNAs are frequently edited by tRNA synthetases [3], which sometimes even edit tRNAs at advanced stages in the translational pathway [4]. Both pre- or post-transfer editing occur. These mechanisms are not exclusive and depend on catalytic sites other than the aminoacylation site [5,6]. The complex editing functions of some tRNA synthetases probably originated from multifunctionality of ancient tRNA synthetases, at the origins of the genetic code and the translational machinery [7]. Note that some mutations affecting editing associate with mitochondrial diseases [8].

## Presentation of the hypothesis

Despite editing, misinsertion sometimes occurs. However, misinsertion is avoided if the misloaded tRNA's anticodon mismatches a codon coding for the misloaded amino acid, a phenomenon potentially meaningful for organisms. If mismatch is unaffected by misacylation, combinatorial probabilities predict their joint occurrence. This phenomenon is called here error compensation. The hypothesis suggested here is that misacylation affects mismatch potentials and hence joint occurrences of misacylations and mismatches do not match combinatorial probabilities, called error coordination. Error compensation is an unavoidable phenomenon whose potential effects are explored elsewhere. Here I focus on error coordination, which is a more readily testable phenomenon because it can be compared to a null hypothesis, that defined by error compensation, hence its detection implies estimating error compensation.

**Figure 1:**
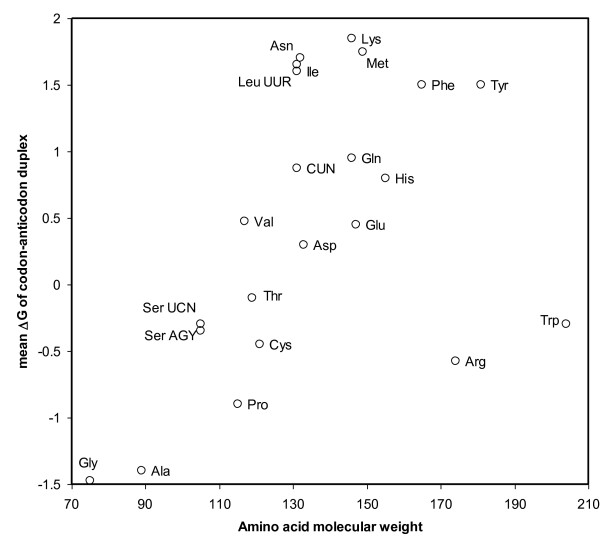
**Duplex stability of codon-anticodons as a function of the molecular weight of their corresponding amino acid**. Stabilities (kilocalories/mol) are averaged across all codons of a given codon family coding for an amino acid.

The tRNA's acceptor stem and the anticodon are not adjacent. Hence properties of amino acids may have little impact on anticodon potentials for interactions with codons. It is nevertheless plausible that such effects exist, even if subtle. These may be larger when the tRNA molecule has relatively low molecular weight (a high pyrimidine content, as uracil and cytosine have lower molecular weights than their purine complements, adenosine and guanine, respectively), and/or is relatively small. Sometimes, small tRNA-like molecules might transfer amino acids, as suggested by the observation of aminoacylated RNA corresponding to the mitochondrial light strand replication origin [9] and hypothetical pre-tRNAs from the primordial RNA world [10]. Misloading a tRNA with an amino acid that drastically differs in size from the cognate amino acid could alter codon-anticodon mismatches, especially for small tRNAs. Misinsertions resulting from such misacylations are probably particularly rare, but would have great impact on protein function [11-14]. Hence error compensation, and even more error coordination, could have significant impact under such conditions. The observation that stabilities of (correctly matched) codon-anticodon duplexes correlate with molecular weights of coded amino acids (Figure 1, stabilities were calculated using Mfold [15,16]) suggests that a constraint between amino acid size and codon-anticodon duplex stability exists. Hence amino acid weight could affect codon-anticodon interactions.

Another amino acid property potentially affecting error coordination is its charge. Charge differences between cognate and misloaded amino acid could affect the tRNA's electro-magnetic properties, including of its anticodon. Codon-anticodon interactions depend on electro-magnetic properties of the interacting atoms (hydrogen atoms with positive partial dipole moments interact with negative partial dipole moments of nitrogen or oxygen in the complementary base). Misacylations altering amino acid charges could affect codon-anticodon interactions: positive charges (arginine, histidine and lysine) might enhance positive partial dipole moments, decrease negative ones; the opposite would occur for negative charges (aspartic and glutamic acids). These effects would alter codon-anticodon duplex stabilities, including for mismatches. Such effects on anticodons are difficult to predict, because tRNA acceptor stems are not adjacent to anticodons. Charge effects on anticodon interactions may be buffered as well as enhanced by particular RNA sequences connecting amino acid and anticodon. Perhaps RNA rich in pyrimidines (which have high dipole moments) functions as conductor of effects of amino acid charge on anticodons. Softwares predicting dipole moments of proteins exist [17,18], hence applications predicting similar properties for loaded tRNAs could be developed on similar principles.

Other amino acid properties might also affect codon-anticodon interactions. This suggests that experimental observations on effects of misacylations on mismatches should be made for all combinations of misacylations and codon-anticodon mismatches, because effects of swapping one amino acid with another may be unpredictable, as different amino acid properties may be relevant. As noted above, specific tRNA sequences may also influence. If this is the case, models predicting these influences would be particularly important for predicting error coordination, because tRNA sequences frequently vary between species and individuals, and it is unrealistic that experimental data will be produced for more than a few model species (most likely *Escherichia coli *and *Homo sapiens*).

## Testing the hypothesis

The comparison between error compensation and coordination is interesting from an epistemological point of view. Error compensation is a trivial phenomenon that necessarily occurs. It can only be quantified and compared between different cases (anticodons, tRNAs, species etc...), so as to test for associations between that quantity and its assumed effects on protein metabolism and related phenomena at higher organismic levels (for different treatments, individuals, species, etc...). Error compensation is a case where a null hypothesis is in itself biologically interesting, similar to the neutral theory of evolution. Error coordination is more adequate in relation to designing classical manipulative experimental tests. It expects that misacylated tRNAs form more stable (or more frequently) mismatched codon-anticodon duplexes with codons coding for the misloaded amino acid than the same tRNA when it is correctly aminoacylated. This can be tested by comparing results from different, independent experiments. Experiments designed to estimate 'competition' between molecules can also be designed. For example, matching and mismatching codons are put in presence of either correctly or incorrectly loaded tRNAs, and duplex stabilities/frequencies of correctly matched and mismatched anticodons are compared (one way competition between codons). Mixing correctly and incorrectly loaded tRNAs with either matching or mismatching codons would estimate one way competition between correctly and incorrectly loaded tRNAs. Two-way competition (all 4 types of molecules are mixed and all duplex types quantified) could also be considered. Ideally, such experiments should be done for all combinations of codons, anticodons and amino acids.

## Implications of the hypothesis

Recent observations suggest that ribosomes select against misacylated tRNAs [19]. Error coordination could be a further mechanism ensuring translational accuracy despite misacylations and mismatches. The translational machinery (the genetic code's amino acid-codon-anticodon assignments, and properties of tRNAs and tRNA synthetases) might be optimized in relation to error coordination if error coordination is observed for a majority of amino acids in relation to either a given tRNA, anticodon, or codon.

Precisely estimating error coordination would imply producing data that include effects of posttranscriptional tRNA modifications [20-22], in particular at wobble positions [23,24], on misacylation and on anticodon-codon interactions. The latter could be used to improve accuracies of RNA secondary structure predictions. Some codons, anticodons, tRNAs, or organisms may more coordinate errors than others. In the former case, selection enhances directly product accuracy, while in the latter it might minimize errors at each single step of the production process. These two types of selections might lead to very different types of genetic, epistatic and metabolic networks. They would probably associate with different types of evolutionary life history strategies (i.e., r- versus K-strategies). In some cases, low error rates in either misacylation or mismatch may result in more misinsertions than higher error rates at both steps, hence selection for error rates above minimal thresholds might occur.

Error compensation and coordination suggest that accurate translation has probabilistic properties. Design of artificial translational systems might take advantage of error compensation and coordination. Primordial systems at the origins of life perhaps used few versatile, yet accurate tRNAs (or tRNA-like molecules), thanks to high error compensation and coordination. Various editing mechanisms [25,26] exist and evolved independently in each tRNA synthetase classes: class I, ValRS, IleRS, and LeuRS (editing presumably evolved in the common ancestor of these enzymes [7]); class II, ThrRS, AlaRS, ProRS, and PheRS [27]. These probably evolved relatively late in the history of the translational machinery [28,29]. Error compensation and coordination probably predates editing mechanisms for these amino acids. Editing mechanisms have not been detected for many tRNA synthetases, and their loss [30,31] in those usually possessing editing capacities suggests that other mechanisms, such as error compensation and coordination, exist and prevent amino acid misinsertion also in modern organisms. Loss of editing functions might associate with increased usages of codons prone to error compensation and coordination, as well as alter tRNA structures if these affect error coordination. Error compensation and coordination are unlikely byproducts of a randomly evolving genetic code and the coevolving translational machinery. They promote translational accuracy in the absence and in addition to editing mechanisms.

## Competing interests

The author declares that he has no competing interests.

## Authors' contributions

HS is the sole author of this publication.
